# Detection of Sialic Acid to Differentiate Cervical Cancer Cell Lines Using a *Sambucus nigra* Lectin Biosensor

**DOI:** 10.3390/bios14010034

**Published:** 2024-01-10

**Authors:** Ricardo Zamudio Cañas, María Eugenia Jaramillo Flores, Verónica Vallejo Ruiz, Raúl Jacobo Delgado Macuil, Valentín López Gayou

**Affiliations:** 1 Laboratorio de Bionanotecnología, Centro de Investigación en Biotecnología Aplicada, Instituto Politécnico Nacional (IPN-CIBA), Tepetitla 90700, Mexico; rzamudioc1700@alumno.ipn.mx (R.Z.C.); rdelgadom@ipn.mx (R.J.D.M.); 2 Laboratorio de Biopolímeros, Escuela Nacional de Ciencias Biológicas, Instituto Politécnico Nacional (IPN-ENCB), Ciudad de México 07738, Mexico; mjarfl@ipn.mx; 3 Laboratorio de Biología Molecular, Centro de Investigación Biomédica de Oriente, Instituto Mexicano del Seguro Social, Metepec 74360, Mexico; veronica_vallejo@yahoo.com

**Keywords:** lectin-based biosensor, attenuated total reflectance Fourier transform infrared (ATR-FTIR), cervical cancer (CC), principal component analysis (PCA)

## Abstract

Pap smear screening is a widespread technique used to detect premalignant lesions of cervical cancer (CC); however, it lacks sensitivity, leading to identifying biomarkers that improve early diagnosis sensitivity. A characteristic of cancer is the aberrant sialylation that involves the abnormal expression of α2,6 sialic acid, a specific carbohydrate linked to glycoproteins and glycolipids on the cell surface, which has been reported in premalignant CC lesions. This work aimed to develop a method to differentiate CC cell lines and primary fibroblasts using a novel lectin-based biosensor to detect α2,6 sialic acid based on attenuated total reflectance Fourier transform infrared spectroscopy (ATR-FTIR) and chemometric. The biosensor was developed by conjugating gold nanoparticles (AuNPs) with 5 µg of *Sambucus nigra* (SNA) lectin as the biorecognition element. Sialic acid detection was associated with the signal amplification in the 1500–1350 cm^−1^ region observed by the surface-enhanced infrared absorption spectroscopy (SEIRA) effect from ATR-FTIR results. This region was further analyzed for the clustering of samples by applying principal component analysis (PCA) and confidence ellipses at a 95% interval. This work demonstrates the feasibility of employing SNA biosensors to discriminate between tumoral and non-tumoral cells, that have the potential for the early detection of premalignant lesions of CC.

## 1. Introduction

Early detection of diseases at their primary stages is crucial to treat the ailment with particular facilities. This is the case of cervical cancer (CC), where the detection of premalignant lesions allows for their efficient removal by surgical treatment [1]. However, CC is in most cases diagnosed at advanced stages, being the second cause of death from cancer in Mexico. So far, cytology and human papillomavirus (HPV) detection are the screening methods in our country for premalignant lesions, and the diagnostic is confirmed with a histopathological study (biopsy), which represents the gold standard [2,3,4,5]. However, cytology lacks the sensitivity (30–80%) to detect premalignant lesions accurately [4,6], because the diagnosis is based on the subjective observation of morphological changes in cervical epithelium cells, which might change depending on the criteria from one observer to another [7,8]. On the other hand, molecular detection of HPV has a restriction to be used as an indicator for premalignant lesions, since young women without cytological alterations can be positive for HPV. Hence, this screening method has been recommended for women older than 30 years, excluding women under this age who would develop alterations that are considered of health relevance.

Therefore, a more effective screening strategy should target a biomarker that expresses the progression of CC from the early stages. A suitable option could be the detection of sialic acid, since cancer progression and premalignant lesions are associated with changes in global sialylation, resulting in an increased expression of the sialic acid on glycoconjugates in malignant cells [9,10,11].

Sialic acid is a family of acidic sugars comprising a nine-carbon backbone that mostly surrounds all cell surfaces by linking to glycoproteins and glycolipids, displaying different functions. The most common sialic acid linkages are α2,3 linked to galactose (Gal), α2,6 linked to Gal and N-acetyl-galactosamine (GalNAc), and α2,8 linked to another sialic acid [11,12]. Taking advantage of this, the interest of this work was the development and study of a lectin-based biosensor that binds preferentially to α2,6 sialic acid to differentiate primary fibroblasts from CC cell lines.

Biosensors are analytical tools that provide a signal throughout the interaction between a composite of a biological recognition element (BRE) and a transducer that will transform the biological signal into a measured value. For several years, biosensors have attracted attention as potential tools for analyzing several environmental, food quality, and medicine molecules [13,14,15].

Biosensors can be classified according to the transducer (optical, electrochemical, piezoelectric, etc.) or the BRE molecules (nucleic acids, proteins, enzymes, antibodies, cells, or biological tissues) [16,17]. In the case of optical biosensors, colloidal biosensors based on gold nanoparticles (AuNPs) are easy to develop, as they do not need hazardous treatments or several steps for their assembly [18].

Optical detection is performed by exploiting the interaction between the optical field and the BRE [17]. The most studied responses used by optical biosensors are surface plasmon resonance (SPR) biosensors, fluorescence chemiluminescence, and surface-enhanced Raman scattering (SERS) [18,19]. Moreover, surface-enhanced infrared absorption spectroscopy (SEIRA)-based biosensors have been reported as a reliable and versatile tool for the identification of medical interest targets using colloidal biosensors [20,21]. The principle of SEIRA is based on signal amplification through the infrared spectrum that can be achieved by the interaction between AuNPs and biological samples using attenuated total reflectance Fourier transform infrared spectroscopy (ATR-FTIR) [22]. Although ATR-FTIR analysis by itself is helpful for the discrimination of cell types and between CC cell lines and healthy cells [23,24,25] and to distinguish premalignant stages [6], it is necessary to target specific biomarkers to develop more sensitive and specific screening responses.

The optical biosensor in this work is proposed as a novel diagnostic tool with high sensitivity and specificity, based on colloidal AuNPs conjugated with *Sambucus nigra* (SNA) lectin. This protein binds with high affinity to sialic acid attached to terminal galactose in α2,6 linkage [11,26].

Herein, we report the application of a novel SNA-based biosensor and the corresponding chemometric analysis to allow for the discrimination between primary fibroblasts and CC cell lines such as HPV-16 positive SiHa, HPV-18 positive HeLa, and HPV-negative C33A. Our results demonstrated that our biosensor not only allowed for discrimination between tumoral and non-tumoral cells but is also capable of clustering, through modifications in the glycosylation pattern of the cell surface of different cell lines used in this study, opening the possibility for its use in the diagnosis of premalignant lesions.

## 2. Materials and Methods

### 2.1. Reagents

All reagents used for the synthesis of AuNPs as well as the HEPES buffer, glacial acetic acid, and SNA lectin were purchased from Sigma Aldrich (St. Louis, MO, USA). Chloroauric acid (HAuCl_4_) used without further purification and medium molecular weight chitosan poly (D-glucosamine) with a degree of deacetylation of 75–85%, were employed for the synthesis of AuNPs. Glacial acetic acid was diluted to 1% aqueous solution before use. All aqueous solutions were made with deionized water.

### 2.2. Assembly of SNA Biosensors

AuNPs were prepared by chitosan reduction in chloroauric acid in a ratio of 7:3 by mixing for 3 h at 80 °C and 650 rpm. Afterward, the solution was centrifuged at 11,000 rpm, and the supernatant was removed and re-suspended in 1 mL of 1 mM buffer HEPES. A volume of 400 µL AuNPs solution was placed in a microtube with 5 µg of SNA lectin, to promote their interaction for 24 h at 14 °C. Then, the mixture was centrifuged at 8000 rpm for 30 min, and the supernatant was removed. The obtained solid was placed on the ATR crystal to collect the FTIR spectrum data. Moreover, characterizations to analyze the construction of the lectin-based biosensor were performed by UV-Vis and dynamic light scattering (DLS) analysis. Furthermore, a Genesys 40 Visible Spectrophotometer (Thermo Scientific, Waltham, MA, USA) and a Zetasizer Nano S-90 (Malvern Instruments, Worcestershire, UK) were used to analyze the construction of the lectin-based biosensor. Transmission electron microscopy (TEM) was performed with JEM2200 (EOL Ltd., Tokyo, Japan) at the Centro de Nanociencias y Micro y Nanotecnologías (Mexico City, México).

### 2.3. Cell lines and Culture

The human CC cell lines SiHa, HeLa, and C33A were obtained from the Centro de Investigación Biomédica de Occidente (CIBO) (Guadalajara, México), kindly provided by Ph.D. Adriana Aguilar Lemarroy from the cell bank of the CIBO Immunology Division [27]. Primary fibroblasts were acquired from the Escuela Nacional de Ciencias Biológicas (ENCB-IPN) (Cdmx, México). The homogeneity of cell culture was verified with RT-qPCR and following the protocol described by Jagadeeshaprasad et al. 2022 [28]. Cell lines and primary fibroblasts were cultured in Dulbecco’s Modified Eagle medium (DMEM) (Gibco, Billings, MT, USA) supplemented with 10% fetal bovine serum (FBS) (Gibco, USA). For the case of fibroblasts, DMEM was supplemented with 15% and 1% antibiotics (penicillin-streptomycin). All cells were incubated at 37 °C in a humidified atmosphere containing 5% CO_2_. Cells were cultured to an 80% confluence and passaged using 1× trypsin with 0.25% ethylene diamine tetraacetic acid (EDTA).

### 2.4. Cell Interaction with SNA Biosensors

Before spectroscopic measurements, all cell lines were detached using 1× trypsin with 0.25% EDTA for 5 min and centrifuged at 2500 rpm for 5 min. After removing the supernatant, cells were washed once with PBS buffer and centrifuged at 2500 rpm for 5 min. Finally, the supernatant was removed, and cell pellets were resuspended in distilled water. The number of cells used in each experiment was 40,000. The solution-containing cells were placed in a microtube and 50 μL of the assembled SNA biosensors were added, to allow for direct interaction for 1 h at room temperature. The solution was centrifuged at 2500 rpm for 5 min, the supernatant was removed, and cells were resuspended with 10 mM HEPES buffer. All samples were manipulated similarly to perform correct comparisons.

### 2.5. ATR-FTIR Spectroscopy Spectra

**Scheme 1:**
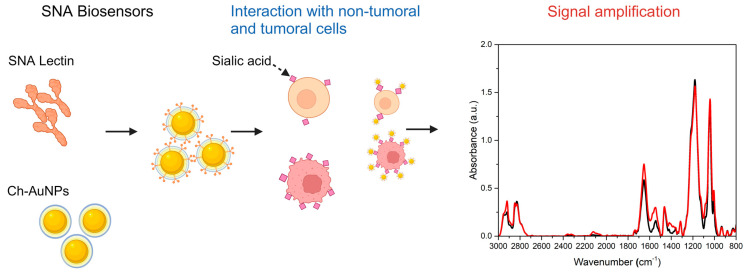
Illustrative diagram of the preparation steps for the detection of non-tumoral and tumoral cells with SNA Biosensors. Scheme 1 was created with BioRender.com.

Spectra were acquired using a Bruker Vertex 70v FT-IR vacuum spectrometer equipped with an A225/Q Platinum ATR unit with a 2.4 single reflection diamond crystal (Bruker Optics, Ettlingen, Germany). For spectra acquisition, 2 µL of the solution was placed on the ATR crystal and dried at room temperature to minimize contributions from water. The crystal was washed with distilled H_2_O before use and between samples. The spectra were obtained in the mid-infrared region (4000–400 cm^−1^), and each spectrum was taken as an average of 132 scans with a resolution of 4 cm^−1^. Measurements were taken in triplicate on three different days to obtain nine spectra for each cell type to ensure cell viability. Scheme 1 illustrates the overall construction of SNA biosensors and the signal amplification resulting from the interaction with tumoral and non-tumoral cells.

### 2.6. Data Analysis

Chemometric analysis, such as PCA, was performed on spectral data to minimize and obtain a new set of orthogonal variables retaining the maximum variability within the data. The new variables were established as components.

PCA analyses were calculated using a full range of the fingerprint register and parts of it. In addition, for standardization data purposes, it was necessary to apply 9-point smoothing, and baseline correction followed by min/max normalization using OPUS v7.0 software. The confidence ellipse around each class represents a 95% confidence interval region. Confidence ellipses can be used as an indicator of certainty about whether the samples truly belong to the specified group. OPUS v7.0 software (Bruker Optics, Ettlingen, Germany), Unscrambler X 10.4, and ORIGIN Pro 2023b (Learning edition) were used for preprocessed data, analysis, and plotting results, respectively.

All data were analyzed using Origin Pro 2023 software to calculate the significance value with the implemented one-way ANOVA using the Tukey test, *p*-values ≤ 0.05 were accepted as statistically significant.

## 3. Results and Discussion

### 3.1. Characterization of SNA Biosensors

Green synthesis of AuNPs using chitosan as a reducing, stabilizing, and functionalizing agent has been broadly described in many works since it possesses reactive hydroxyl and amine groups that can be used for the immobilization of biomolecules [29,30,31]. The electronegativity property of chitosan makes it suitable for synthesis whereas its functional groups NH_2_ and OH are responsible for reducing the Au precursor [32,33,34]. Furthermore—NH_3_^+^ groups stabilize AuNPs [34]. It is known that Au (III) reduction in chitosan solution can be considered a three-stage process that leads to the hydrolysis and chain fragmentation of chitosan, and its products act as the main reducing species [32].

**Figure 1:**
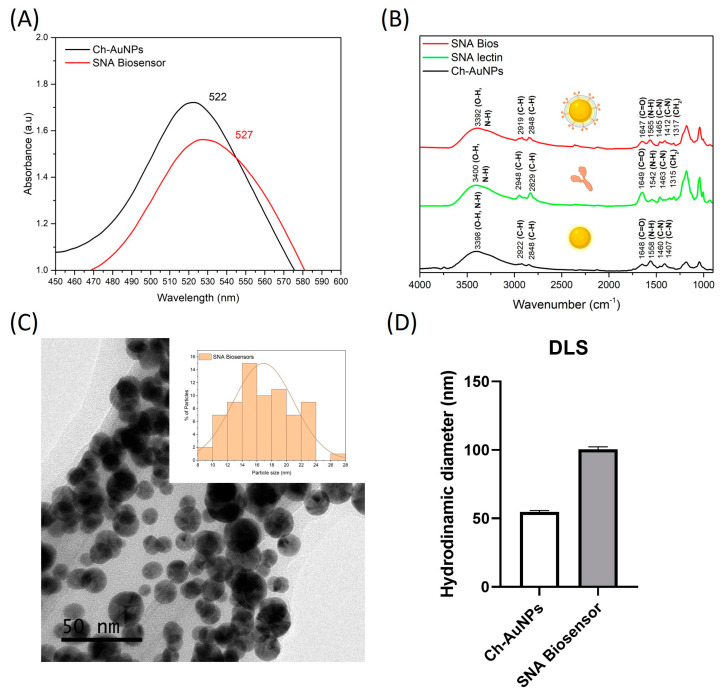
UV-Vis spectra of Ch-AuNPs (black) and conjugated with SNA lectin (red) (**A**). ATR-FTIR spectra of Ch-AuNPs (black), SNA lectin control (green), and SNA biosensors (red) (**B**). Representative TEM micrographs of SNA biosensors and their distribution histogram (**C**). Hydrodynamic results of Ch-AuNPs and SNA biosensors (**D**). Figure 1B was created with BioRender.com.

Chitosan-capped gold nanoparticles (Ch-AuNPs) were successfully synthesized according to the obtained UV-Vis spectra, displayed by a characteristic ruby red color and a maximum absorption band around 522 nm. This result is similar to other works [30,35] and consistent with the SPR [36,37]. The next step was the conjugation with SNA lectin which produced a shift in the maximum absorbance peak of 5 nm (522 to 527), observed in Figure 1A. Since SPR is sensitive to changes in the refractive index caused by interactions between the sensor surface and other molecules, the shift to longer wavelengths was useful to confirm the conjugation of Ch-AuNPs and SNA lectin [31,38,39].

From ATR-FTIR results, we compared the spectra of Ch-AuNPs, SNA lectin, and SNA biosensors. Ch-AuNPs showed peaks at 3398, 2922, 1648, 1558, 1460, and 1410 cm^−1^, displaying signals from pure chitosan at 3398, 2922, 2848, 1648, 1558, and 1410 cm^−1^. Peaks are associated with stretching vibrations overlapping between O–H and N–H (3398 cm^−1^), stretching vibrations of C–H (2922 and 2848 cm^−1^), stretching vibrations of C=O from carbonyl groups (1648 cm^−1^), and C–N and N–H bending vibrations in amide III and II (1558 and 1410 cm^−1^, respectively) [32,34]. After conjugation with SNA lectin, SNA biosensors spectra displayed peaks at 1647, 1565, and 1317 cm^−1^, corresponding to N–H bending and C–N stretching vibrations, N–H bending and C=O stretching vibrations associated with amide I, II, and III peaks [40], displayed also on the SNA pure lectin spectra (Figure 1B, green). The observed changes in the band positions from SNA biosensors compared to Ch-AuNPs and pure lectin are related to changes in the refractive index due to biomolecular interactions [41]. Since greater shifts were not observed, we conclude that there are no modifications to the lectin’s structure and conformation, an important factor in maintaining the binding site from lectin.

SNA biosensors were further characterized by TEM analysis to verify the nanoparticles’ size and shape. Although there are ways to indirectly know the average size of nanoparticles using UV-Vis results like the Haiss equation [42], it is essential to know not only size but shape, which will impact the optical and conjugation properties, such as the surface area that will enable the surface of the nanoparticles to be coated with hundreds of molecules [43]. The best way to achieve this is by using TEM. TEM image of Ch-AuNPs (Figure 1C) illustrates that most particles have a spherical shape, with an average diameter of 16 nm. Following the results, DLS was used to verify conjugation, as it is a powerful method for determining the hydrodynamic sizes of nanoparticles [44,45].

The hydrodynamic size of SNA biosensors measured by DLS was 100 nm (Figure 1D), a larger size than that obtained by TEM (16 nm). This difference in size is because TEM gives a single measurement from the solid AuNPs core, neglecting the surface charge of the functional groups of chitosan and lectins. Hence, the hydrodynamic size of Ch-AuNPs was 50 nm, correlating with those reported by other works [32,45], also the polydispersity index was 0.413. DLS analyses demonstrated that SNA biosensors were properly conjugated since the hydrodynamic diameter was higher than that obtained from Ch-AuNPs (50 to 100 nm) [46].

### 3.2. Infrared Spectroscopy

**Figure 2:**
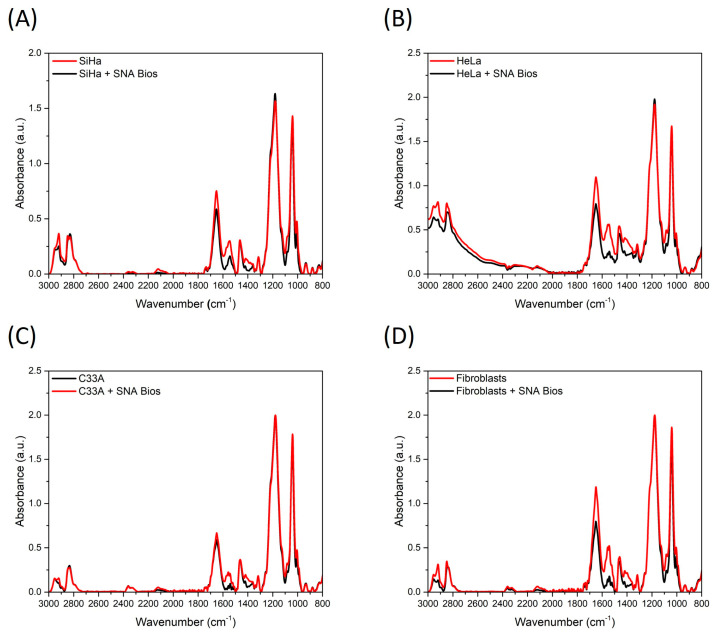
Mean ATR-FTIR spectra (baseline corrected and min/max normalized) of control cells and after interaction between SNA biosensors and (**A**) SiHa; (**B**) HeLa; (**C**) C33A; and (**D**) primary fibroblasts.

ATR-FTIR was used to analyze the interaction between SNA-based biosensors and cells like primary fibroblasts and CC cell lines, HPV-16 positive SiHa, HPV-18 positive HeLa, and HPV negative C33A. After the interaction, spectra were taken and compared with spectra from each cell line as a control reference (Figure 2A–D), where nine spectra were collected from each measurement. Similar peaks are displayed in all cell lines, showing characteristic IR absorption peaks associated primarily with C–H from lipids (2950–2900 and 2800 cm^−1^), as well as amide I (1650 cm^−1^), amide II (1550 cm^−1^), asymmetric and symmetric methyl bending modes (1470–1400 cm^−1^), and stretching vibrations of C–H bonds (1360–1300 cm^−1^) for the fingerprint region [47,48]. Noticeably, two prominent peaks were observed at 1200 and 1000 cm^−1^, related to the HEPES buffer employed to maintain cell stability covering signals associated with sialic acid such as 1238, 1210, 1142, 1125, 1068, and 1024 cm^−1^ [49]. Nevertheless, a signal enhancement is observable in all detections at the 3000–2800 and 1650–1350 cm^−1^ intervals.

**Figure 3:**
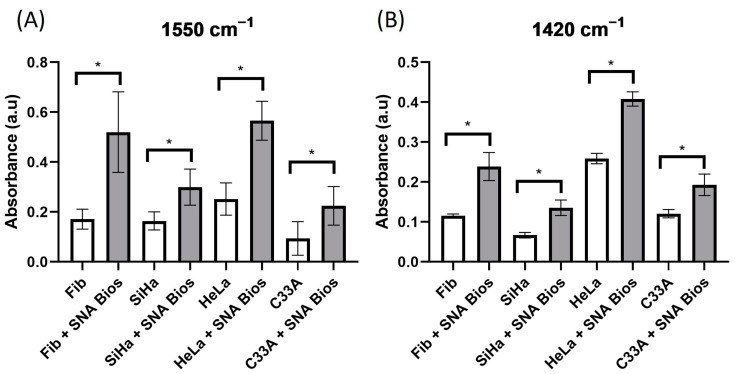
ANOVA (Tukey, *p* > 0.05) of maximum absorbance peak at (**A**) 1550 cm^−1^; (**B**) 1420 cm^−1^ of control cells (white bars) and after interaction with SNA biosensors (gray bars). Star * denotes that the differences are statistically significant.

This phenomenon is attributed to the SEIRA effect, as reported in other works [20]. Signal enhancement will be specific according to the target molecules. The obtained peaks at ≈1650, ≈1550, ≈1418, and ≈1398 cm^−1^ correspond to carboxyl groups in amide I (1650 cm^−1^), vibrations of N–H and C–N linkages of N-acetyl groups and N-acyl groups in amide II (1550 cm^−1^), and vibrations of N–H and C–N groups in amide III (1400 cm^−1^), which are enhanced and reported for sialic acid [50,51,52]. It has been described that SEIRA enhancement is conditioned to the metal type, shape, and surface of nanostructures, and the substrates used for deposition [53,54]. In our particular case, we employed colloidal biosensors using AuNPs, which have lower amplifications than other shapes (stars) [55], but the obtained SEIRA effect is strong enough to observe and analyze differences. According to this, several reports confirm that the SEIRA effect will have lower amplifications (up to 10^4^) in comparison with the SERS effect (up to 10^14^) [56,57]. Our developed SNA biosensor achieved a signal enhancement of detection of three-fold in the signal at 1550 cm^−1^ (Figure 3A) and up to two-fold in the band at 1420 cm^−1^ (Figure 3B). We did not consider the peak at 1650 cm^−1^ to avoid any influence of potential water peaks [24].

Differences between the enhancement results might be attributed to how biosensors interact with α2,6 sialic acid on the cell surface. The distance between the transducer and the target analyte is essential for signal enhancement [31]. On the other hand, CC cell lines are expected to have higher concentrations than non-tumoral cells [58]; however, we believe that due to the shape and size of our particles, along with the presence of more than one lectin molecule on its surface, they may be binding to more than one site, interfering with the binding of more sensors on the cell surface. To provide a deeper analysis, we performed PCA and confidence ellipse analyses from our resulting spectra, since it is a powerful tool for the interpretation of spectral data, reducing the information into new variables or components that explained the variability of the experimental data with higher accuracy [25,59].

### 3.3. Discriminant Analysis

**Figure 4:**
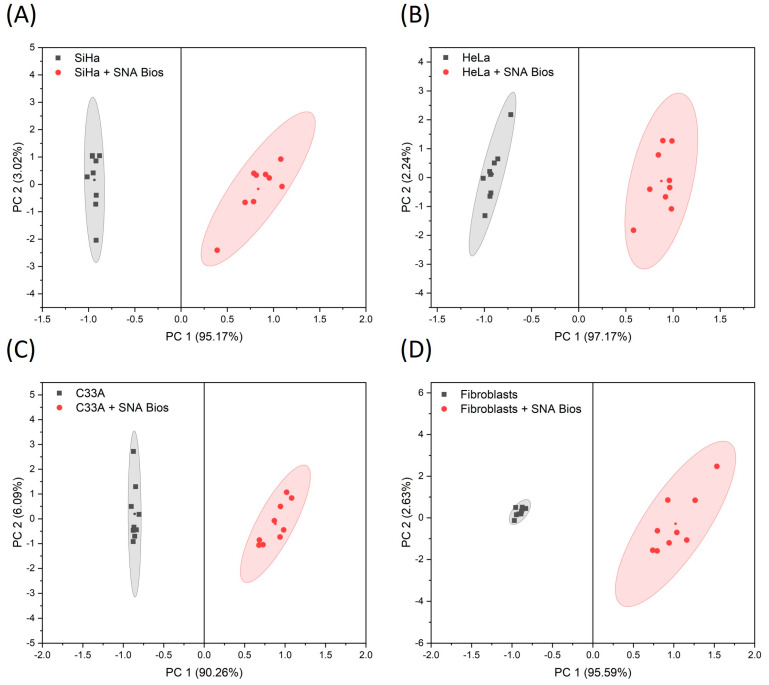
Score plots from PCA analysis (PC1 vs. PC2) and 95% confidence ellipses in the spectral region (1500–1350 cm^−1^) from detections with SNA biosensors in (**A**) SiHa; (**B**) HeLa; (**C**) C33A; and (**D**) primary fibroblasts.

Dimension reduction and multivariate statistical methods, such as PCA and confidence ellipses were useful tools to further differentiate the results obtained from ATR-FTIR spectra after detection with SNA biosensors over primary fibroblasts and the CC cell lines. A 2D score plot was generated using different spectral ranges from the whole spectrum, emphasizing the region where the SEIRA effect displays differences. As expected, the 1500–1350 cm^−1^ spectral range allowed for discriminations in all samples. From the constructed PCA model, we obtained two principal components (PCs), accounting for more than 90% of the total variations of the linear system in all numerical analyses: SiHa (98%), HeLa (99%), C33A (96%), and primary fibroblasts (97%) (Figure 4A–D). All of the four samples of cells detected with SNA biosensors were identified and clustered in the positive axis of PC1, compared with the control cells that clustered in the negative axis of PC1. Moreover, the ellipse around each cluster also represented a 95% confidence interval region. Gajjar et al. [6] reported that “confidence ellipse/confidence region can be used to indicate the reliability of the spectral category means”. Since the generated ellipses are properly separated and do not overlap, we can demonstrate precision in the clustering to classify them [60,61].

**Figure 5:**
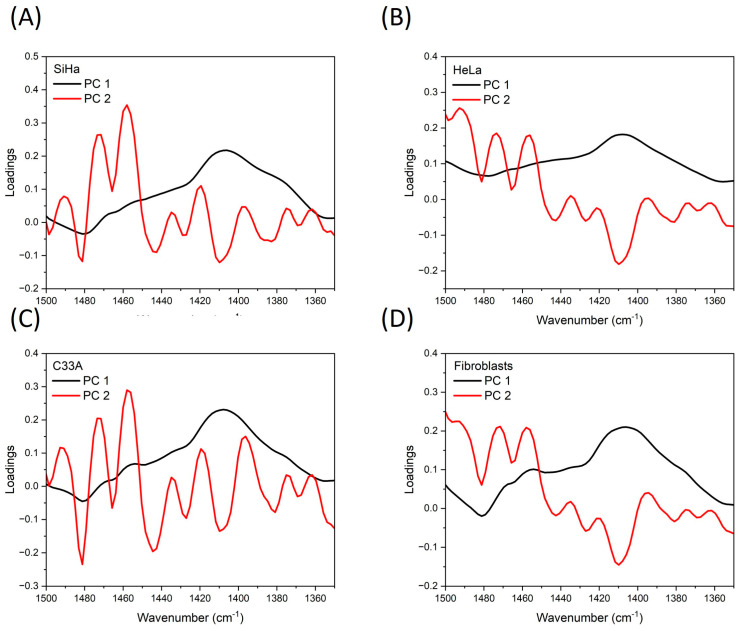
Loading plots from PCA analysis (PC1 vs. PC2) in the spectral region (1500–1350 cm^−1^) from detections with SNA biosensors in (**A**) SiHa; (**B**) HeLa; (**C**) C33A; and (**D**) primary fibroblasts.

**Figure 6:**
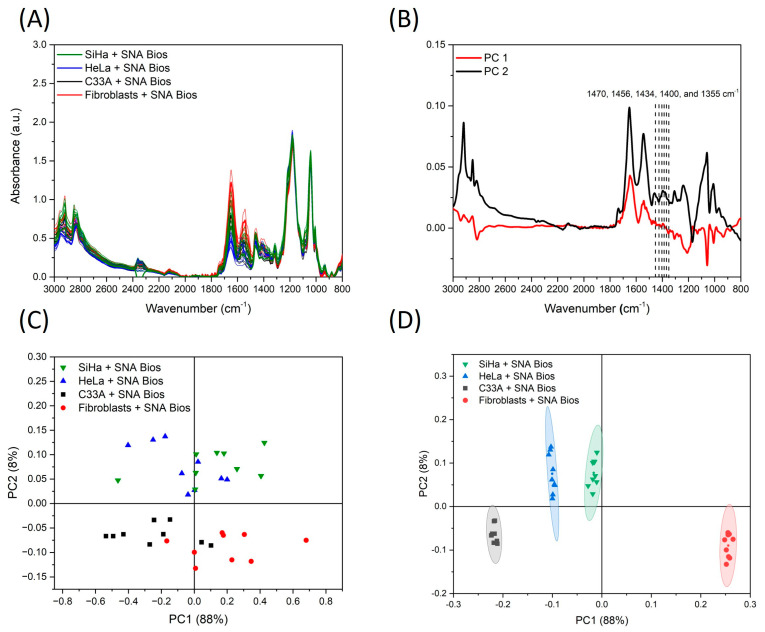
(**A**) ATR-FTIR spectra (baseline corrected and min/max normalized) of detections with SNA biosensors; (**B**) loading plot using the whole spectrum (4000–400 cm^−1^) of detection with SNA biosensors; (**C**) score plot using peaks within the range of 1500–1350 cm^−1^; and (**D**) score plot with 95% ellipse confidence using peaks within the range of 1500–1350 cm^−1^.

Afterward, analyzing loading plots helps to relate peaks to each obtained component [62,63]. From the PC1 loading plot, peaks at ≈1480, ≈1450, and ≈1400 cm^−1^ account for the maximum variance and are related to sialic acid signals [50,64,65], which agree with peaks enhanced by the SEIRA effect. Regarding PC2, peaks at 1490, ≈1480, ≈1470, ≈1455, ≈1420, ≈1400, ≈1390, and ≈1380 cm^−1^ account for the maximum variance (Figure 5A–D). These findings confirmed that clustering results from the SEIRA effect were produced by detecting sialic acid on the cell surface. The next step of the analysis was to determine if results from detections helped to discriminate primary fibroblasts from CC cell lines. A new data set was constructed using only the spectra from the obtained detection results (Figure 6A). Then, the PCA model was constructed using all the spectra data (4000–400 cm^−1^). From the loading plot, we identified five potential peaks to cluster samples at ≈1470, 1456, 1434, 1400, and 1355 cm^−1^ (Figure 6B) which correspond to symmetric and asymmetric methyl bending modes (1450–1400 cm^−1^), and C–N, N–H bending vibrations in amide III (1400–1300 cm^−1^), [47,48]. These peaks include those associated with sialic acid and are the same as those previously reported for the SEIRA enhancement [50,64,65].

Finally, a new PCA model was recalculated using peaks from the loading plot obtained from the previous PCA model (1470, 1456, 1434, 1400, and 1355 cm^−1^) associated with sialic acid [50,64,65]. This recalculation allowed for the clustering of samples between classes (Figure 6C). From the two first components, we achieved more than 90% of the explained variance (88% for PC1 and 8% for PC2). We observed samples clustered on different quadrants according to their cell type, but some others were overlapped (Figure 6C). Nevertheless, after applying confidence ellipses, the samples were clustered without overlapping. Primary fibroblasts were clustered on the positive axis from PC1, while CC cell lines clustered on the negative axis of PC1 (Figure 6D), thus confirming that PC1 is associated with characteristics from tumoral and non-tumoral cells, as well as related with differences in the concentration of sialic acid. The properly separate confidence ellipses illustrate the efficacy of PCA algorithms to differentiate primary fibroblasts and CC cell lines with a 95% confidence interval region (Figure 6D). Furthermore, the SEIRA effect produced by SNA biosensors is strong enough to achieve these results.

From the literature, it was found that the combination of spectral data with PCA analysis and confidence ellipses has been used to study diseases such as bladder, prostate, and breast cancer [66,67,68].

## 4. Conclusions

Herein, a novel SNA biosensor is proposed to discriminate tumoral and non-tumoral cells by detecting α-2,6 sialic acid on the cell surface using spectral data. ATR-FTIR results showed a signal enhancement associated with the SEIRA effect, caused by the interaction of the biosensor with sialic acid. These amplifications were further analyzed with chemometric analysis using PCA and confidence ellipses.

The PCA model using spectral data showed differences between spectra from primary fibroblasts and CC cell lines, with a clear distinction primarily due to the PC1 score. The loading plot of PC1 confirmed that peaks related to sialic acid at 1470, 1456, 1434, 1400, and 1350 cm^−1^ were the significant sources of discrimination between samples. The confidence ellipse classified them into four main groups with a 95% confidence interval.

These preliminary findings support the potential application of SNA biosensors with chemometric analysis and confidence ellipses to distinguish tumoral and non-tumoral cells. This is a proof-of-concept biosensing strategy that requires further and more extensive studies to expand the possibility of implementing this method in clinical laboratory diagnostics to detect premalignant lesions of CC.

## Data Availability

Data will be made available on request.
